# Author Correction: Engineering of high-precision base editors for site-specific single nucleotide replacement

**DOI:** 10.1038/s41467-019-10069-4

**Published:** 2019-05-01

**Authors:** Junjie Tan, Fei Zhang, Daniel Karcher, Ralph Bock

**Affiliations:** 1 Max-Planck-Institut für Molekulare Pflanzenphysiologie, Am Mühlenberg 1, 14476 Potsdam-Golm, Germany; 2 Present Address: National Key Laboratory of Crop Genetic Improvement, Huazhong Agricultural University, 430070 Wuhan, Hubei China

**Keywords:** Genetic engineering, CRISPR-Cas9 genome editing, Targeted gene repair

Correction to: *Nature Communications* 10.1038/s41467-018-08034-8, published online 25 January 2019.

The original version of this Article contained an error in Fig. 5. In the panel c, the right bar chart was inadvertently replaced with a duplicate of the left bar chart. This has been corrected in both the PDF and HTML versions of the Article.

