# Supplementary material for: Engineering of high-precision base editors for site-specific single nucleotide replacement
Source: Nat Commun. 2019 Jan 25;10:439. doi: 10.1038/s41467-018-08034-8 (PMC6347625; doi:10.1038/s41467-018-08034-8)
Supplement: Supplementary file 1 — Supplementary Information [file 41467_2018_8034_MOESM1_ESM.pdf]

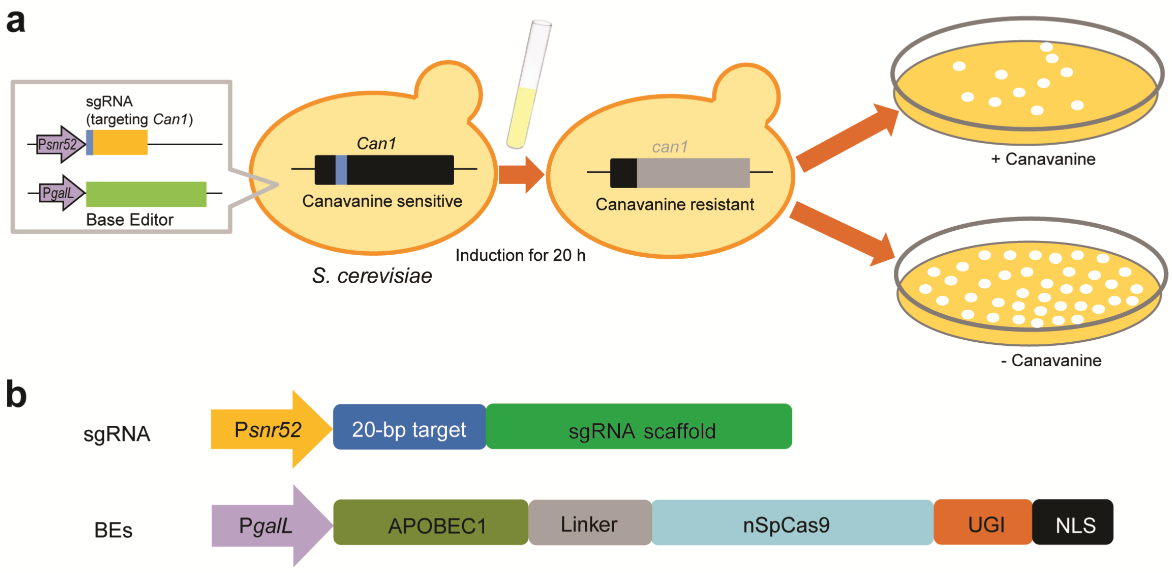

c

BE	Linker	Number of amino acids
BE3	SGSETPGTSESATPES (=XTEN)	16
BE-NL	No linker	0
BE-PAP	PAP	3
BE-PAPAP	PAPAP	5
BE-PAPAPA	PAPAPA	6
BE-PAPAPAP	PAPAPAP	7
BE-PAPAPAPA	PAPAPAPA	8
BE-P(AP)4	P(AP)4	9
BE-P(AP)7	P(AP)7	15
BE-P(AP)10	P(AP)10	21
BE-A(EAAAK)2A	A(EAAAK)2A	12
BE-A(EAAAK)3A	A(EAAAK)3A	17

Supplementary Figure 1 Canavanine selection and construction of base editors with different linkers between the deaminase domain and nCas9. **a** Use of canavanine selection to determine base editing efficiency. Vectors expressing the base editor and the sgRNA are co-transformed into yeast cells. Transgene expression was induced for 20 h before plating of culture aliquots on YPAD rich medium with (+ Canavanine) or without L-canavanine (- Canavanine). The colony count on + Canavanine plates divided by the colony count on - Canavanine plates served as a measure of editing frequency. **b** Design of base editor and sgRNA constructs. The

vector backbones are derived from p415 and p426 (https://www.addgene.org/43804/; see Methods). The sgRNAs are expressed under the snoRNA *SNR52* promoter, expression of the base editors is driven by the inducible *GalL* promoter. nSpCas9: *Streptococcus pyogenes* Cas9 nickase. **c** List of linkers used in the BE constructs. Amino acid sequence and length of all linkers are given.

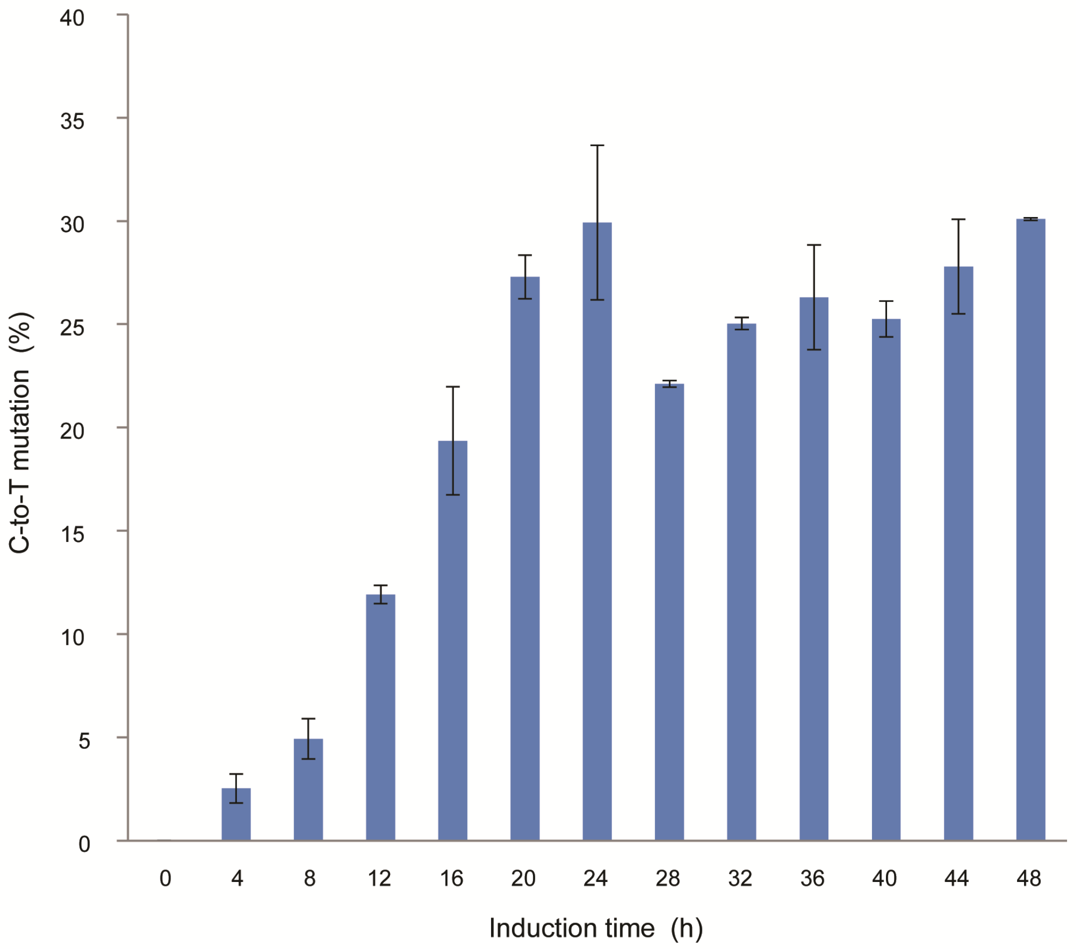

Supplementary Figure 2 Effects of the induction time on the mutation frequency caused by base editors. Plasmids expressing BE3 and an sgRNA targeting site *Can1-5* (Fig. 1a) were co-transformed into yeast cells. BE expression was induced for the times indicated prior to plating on medium with or without canavanine (Supplementary Figure 1a). The C-to-T mutation frequency represents the ratio of the colony count on canavanine-containing plates and the colony count on canavanine-free plates. An induction time of 20 h is sufficient in that longer times do not substantially increase the editing frequency. Values and error bars reflect the mean and standard deviation of three independent biological replicates.

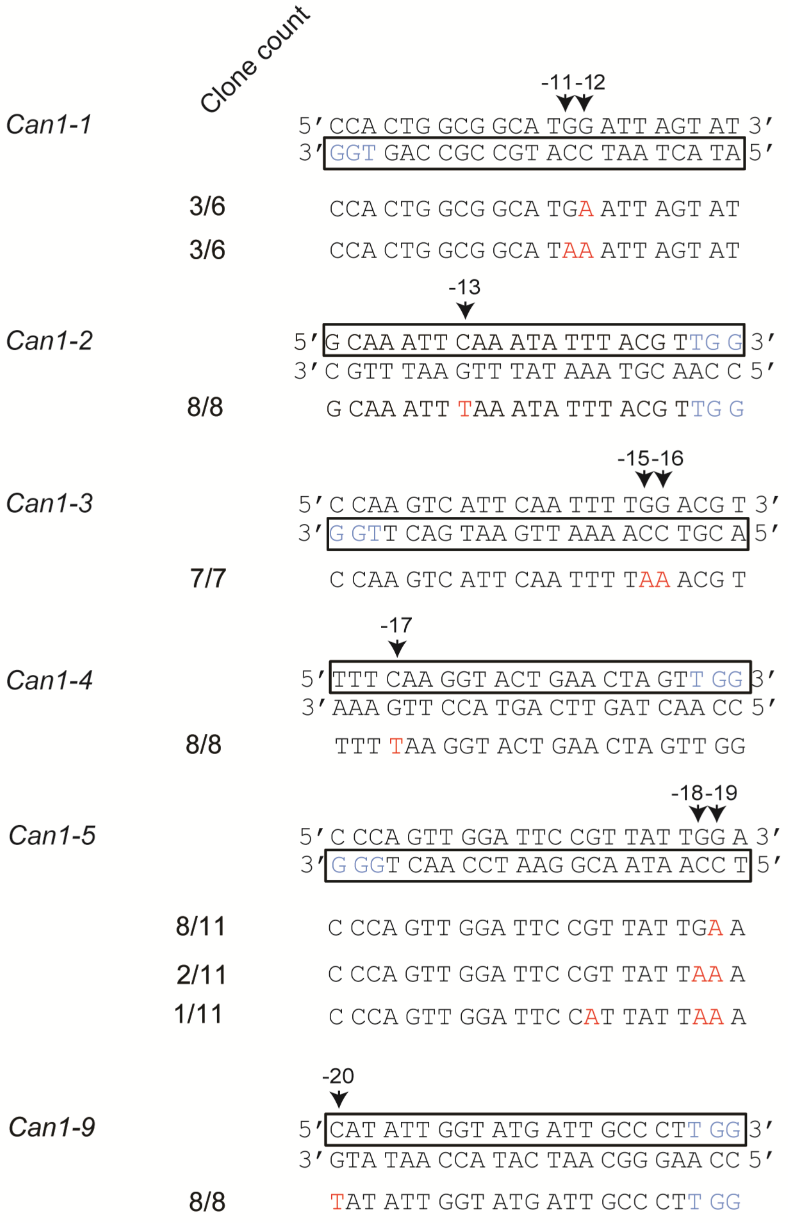

Supplementary Figure 3 Alignment of mutated sequences with the corresponding target sequences in the *Can1* locus. The editing assays were conducted in yeast strains expressing BE3 and of the respective sgRNA. The clone count indicates the number of clones with a specific mutation pattern over the total number of randomly picked canavanine-resistant colonies. The target regions were PCR-amplified and sequenced. The unmutated reference sequences are shown with the target site boxed and the PAM sequence in blue. Arrowheads indicate positions (relative to the PAM) of targeted bases within the protospacer that cause *Can1* inactivation upon mutation from C to T. Note that all analyzed canavanine-resistant colonies undergo at least one C-to-T conversion event within the target region.

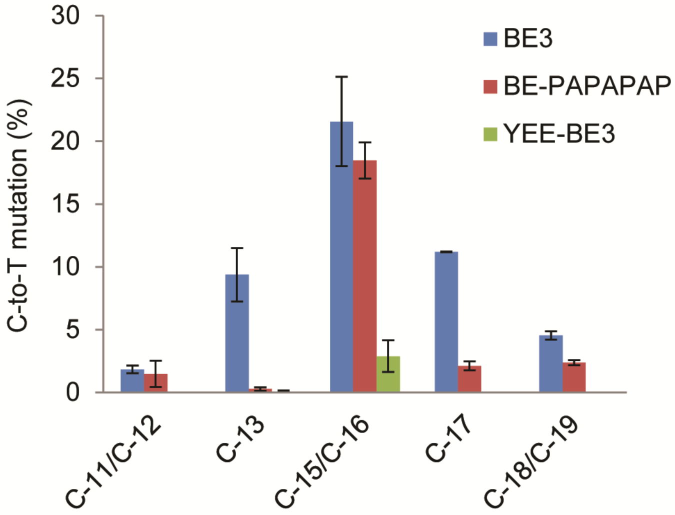

Supplementary Figure 4 Comparison of base editing outcome of BE3, BE-PAPAPAP and YEE-BE3. The x-axis represents the target Cs within the protospacers. The y-axis shows their C-to-T editing frequency (see Methods and Supplementary Figure 1). Sequences of canavanine-resistant mutants aligned with the corresponding reference sequences are shown in Supplementary Figure 3. Values and error bars represent the mean and standard deviation of three independent biological replicates.

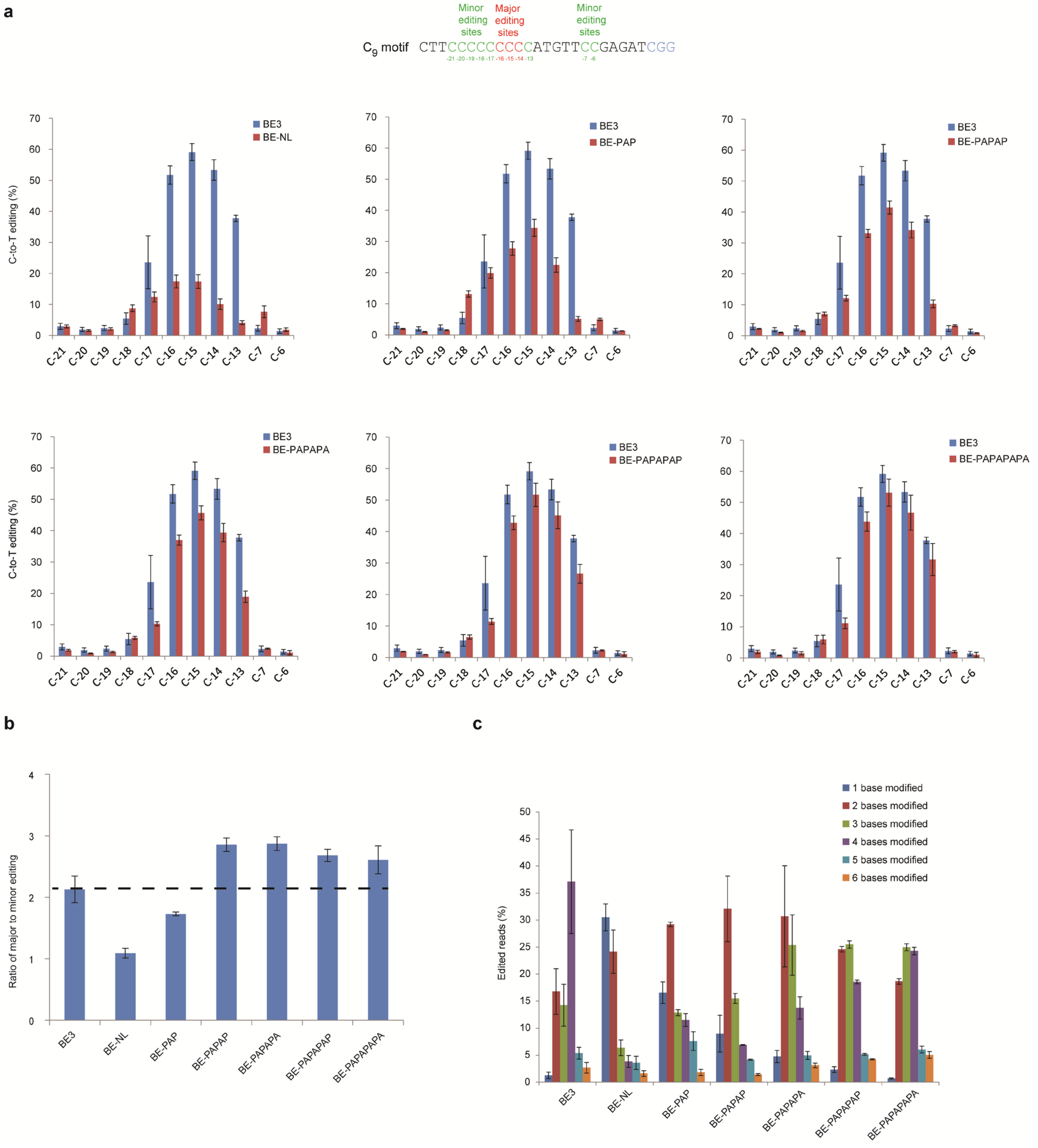

Supplementary Figure 5 Base editors with 5-8 amino acid rigid proline-rich linkers exhibit narrowed editing windows. **a** Comparison of the widths of the editing window of BE3, a linkerless BE (BE-NL; Supplementary Figure 1) and five BEs with short rigid linkers. The sequence of the target (C)9 motif in the protospacer is shown with the major editing sites (-14 to -16) in red and the minor editing sites (-21 to -17, -13, -7 and -6) in green. **b** Ratios of editing at major sites to editing at minor sites as an indicator of the site selectivity of base

editing. **c** Product distribution of base editing. The products of BE3 editing mainly contain four simultaneously edited nucleotides, whereas base editors with short rigid linkers are significantly more specific and predominantly contain 2-3 edited positions. % of edited reads represents the percentage of the total edited reads that contain the products shown. Values and error bars reflect the mean and standard deviation of three independent biological replicates.

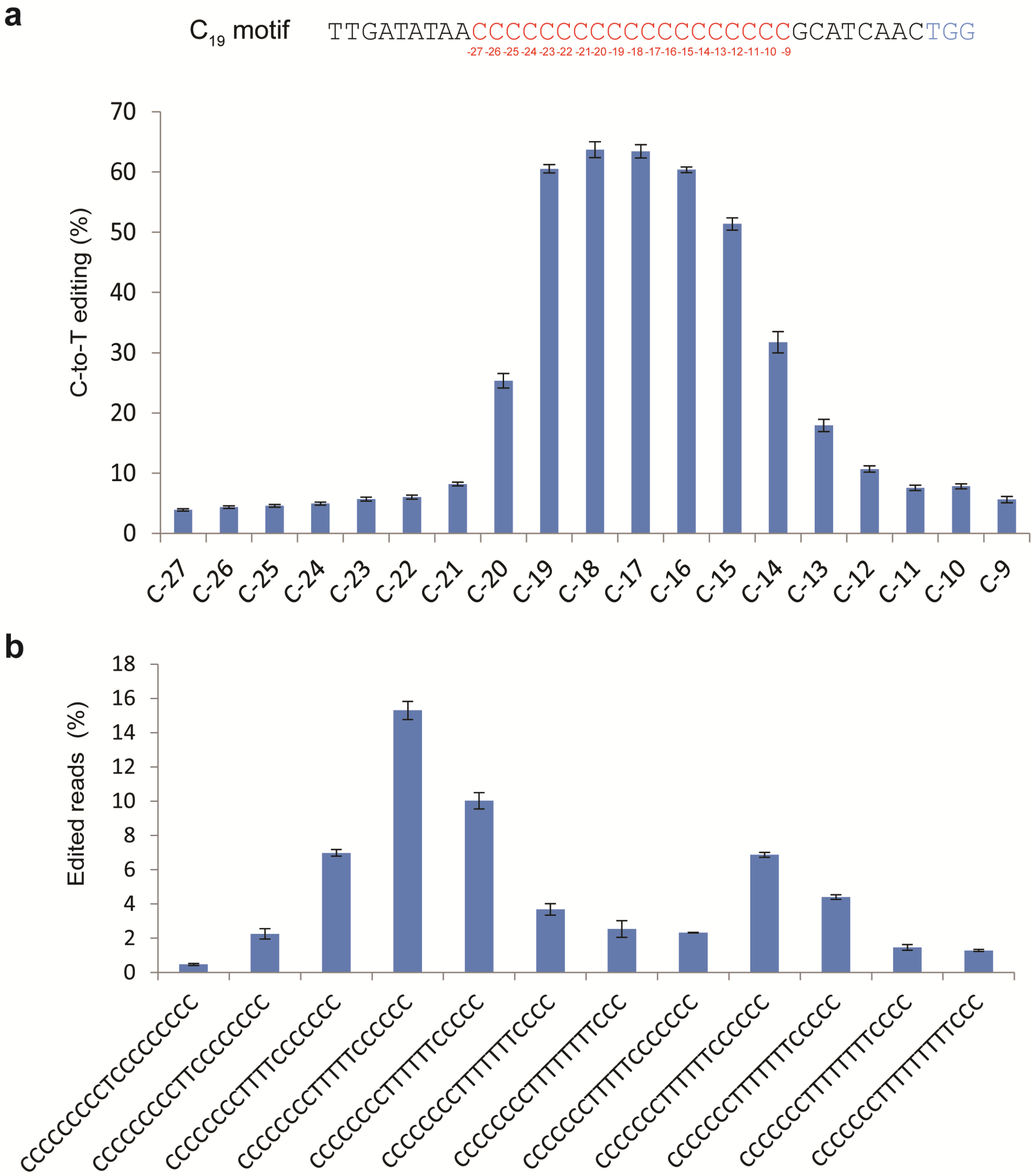

Supplementary Figure 6 Base editing outcome of nCDA1-BE3 targeting a $(C)_{19}$ motif. **A** Editing frequency of each C. The $(C)_{19}$ sequence is shown with numbers representing the position of each C relative to the PAM (blue). **b** Distribution of the 11 main editing products in comparison to the C-18 edited product (first bar). Edited reads (%) is the percentage of the total edited reads that represent the products shown. The 12 products account for 57.62% of the edited reads. Values and error bars represent the mean and standard deviation of two independent biological replicates.

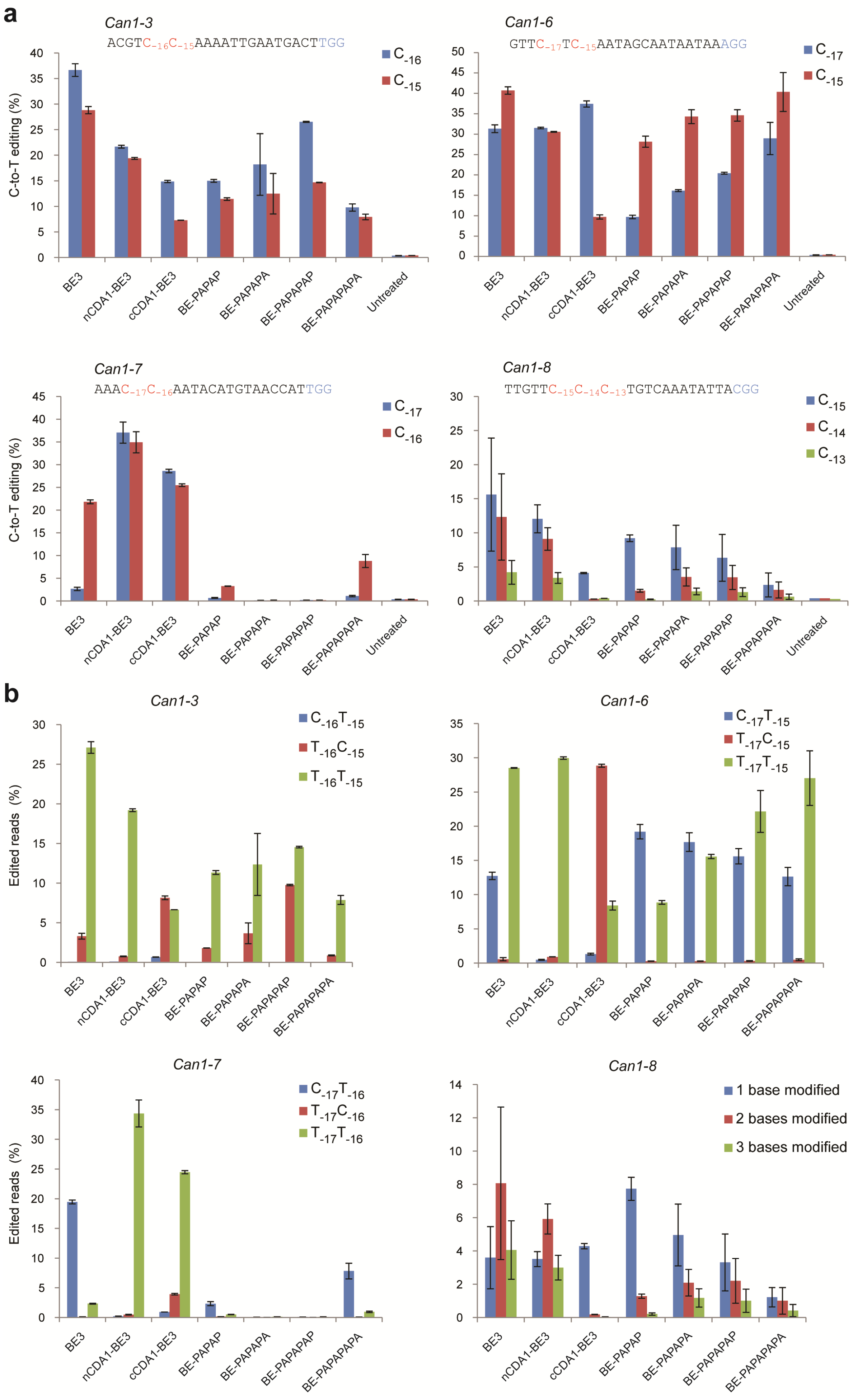

a
Can1-3
ACGTC-16C-15AAAATTGAATGACTTGG
C-16
C-15
C-to-T editing (%)
BE3
nCDA1-BE3
cCDA1-BE3
BE-PAPAP
BE-PAPAPA
BE-PAPAPAP
BE-PAPAPAPA
Untreated
Can1-6
GTTC-17TC-15AATAGCAATAATAAAGG
C-17
C-15
Can1-7
AAAC-17C-16AATACATGTAACCATTGG
C-17
C-16
Can1-8
TTGTTC-15C-14C-13TGTCAAATATTACGG
C-15
C-14
C-13
b
Can1-3
C-16T-15
T-16C-15
T-16T-15
Edited reads (%)
Can1-6
C-17T-15
T-17C-15
T-17T-15
Can1-7
C-17T-16
T-17C-16
T-17T-16
Can1-8
1 base modified
2 bases modified
3 bases modified

Supplementary Figure 7 Capability of different base editors to distinguish between adjacent Cs. **a** Base editing outcome of BE3, nCDA1-BE3, cCDA1-BE3 and selected BEs with rigid proline-rich linkers (Fig. 1; Supplementary Figures 1 and 2). The target loci contain multiple Cs within the protospacer. At sites *Can1-3*, *Can1-6* and *Can1-8*, cCDA1-BE3 and BEs with rigid linkers show increased preference for a specific cytidine. At *Can1-7*, APOBEC1-based editors show a strong preference for C_{-16} over C_{-17}, whereas CDA1 base editors exhibit comparable efficiency for both Cs. **b** Product distribution of base editing. At sites *Can1-3*, *Can1-6* and *Can1-8*, cCDA1-BE3 and BEs with short rigid linkers display substantially more singly modified products than BE3, even when adjacent Cs are present. At *Can1-7*, APOBEC1 base editors produce predominantly singly modified products in comparison to CDA1 base editors, which mainly produce two simultaneous modifications. % of edited reads represented the percentage of the total sequencing reads that contain the products shown. Values and error bars represent the mean and standard deviation of two independent biological replicates.

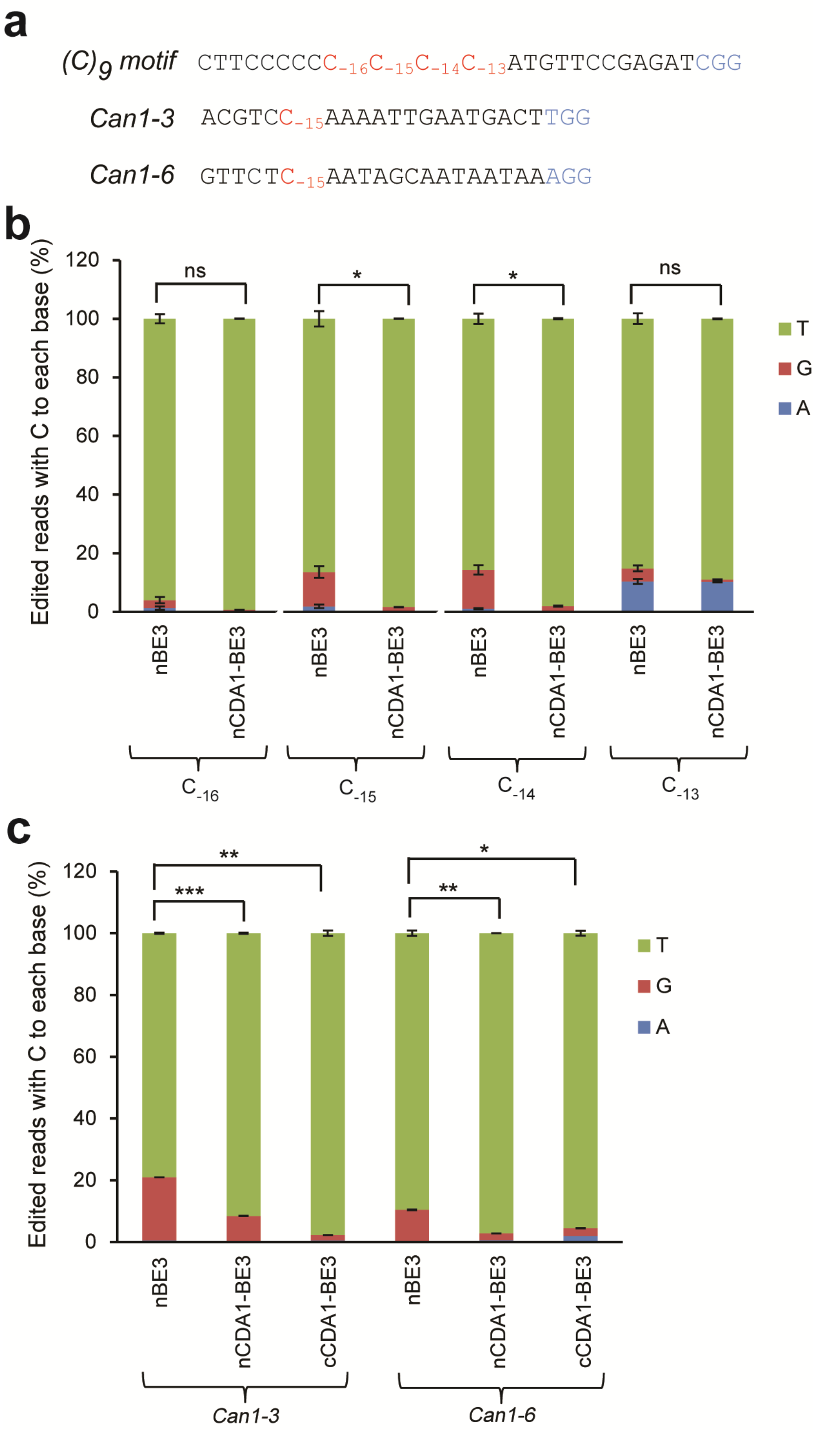

Supplementary Figure 8 CDA1 base editors increase the product purity compared to BE3 editors. **a** Protospacers and PAM (blue) sequences of genomic loci tested, with the target Cs shown in red. **b** Product distribution among edited $(C)_9$ sequencing reads (reads in which the target C was mutated) for nBE3 and nCDA1-BE3. **c** Product distribution among edited *Can1-3* and *Can1-6* sequencing reads for nBE3, nCDA1-BE3 and cCDA1-BE3. Values and error bars represent the mean and standard deviation of two biological replicates. ns: $p \geq 0.05$; *: $p < 0.05$; **: $p < 0.01$; ***: $p < 0.001$ (by two-tailed Student's t-test).

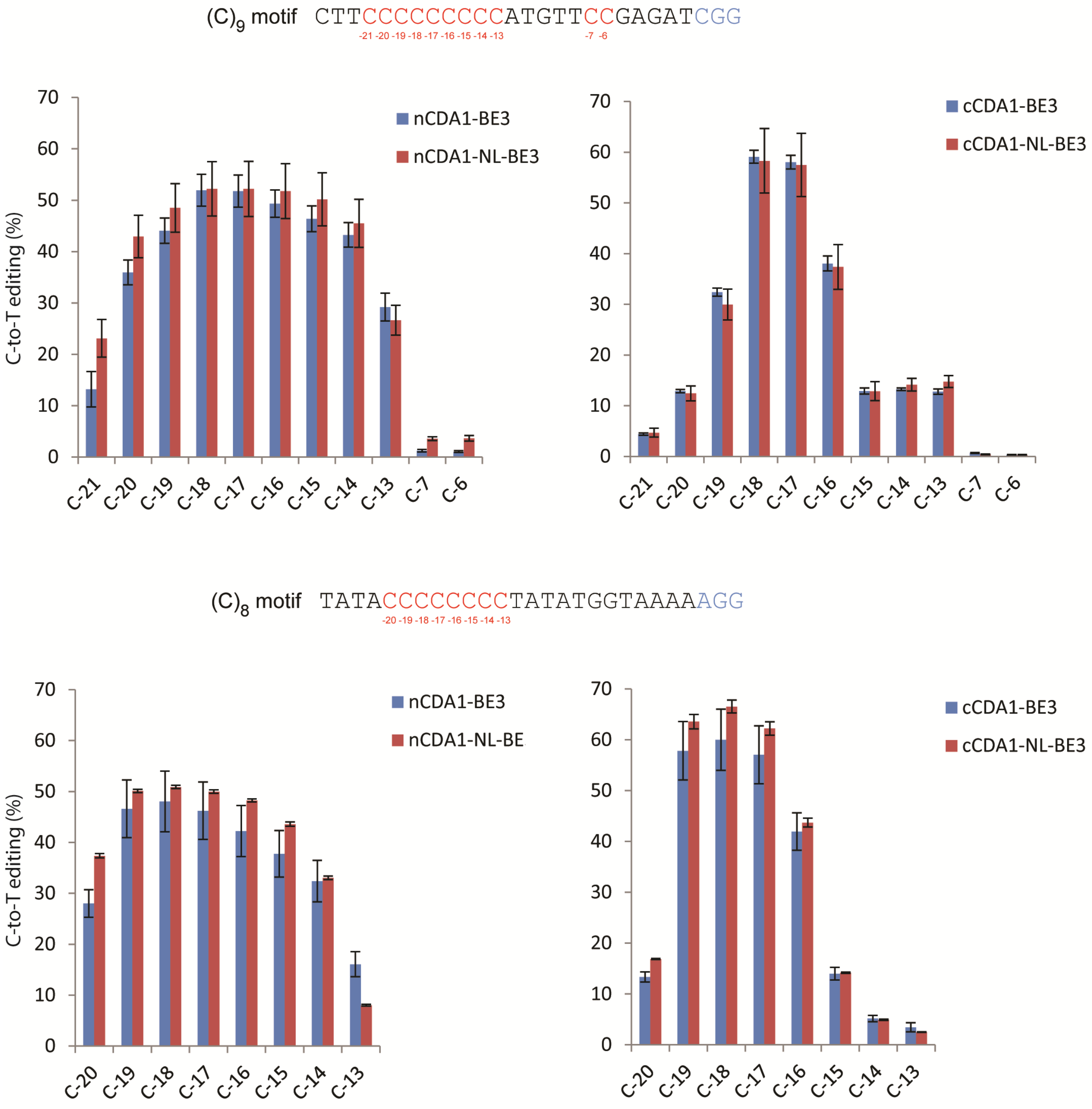

Supplementary Figure 9 Removal of the linker between CDA1 and nCas9 does not alter the width of the editing window or the editing efficiency of either N-terminal or C-terminal fusions. The two target sequences are shown with the target Cs in red and their positions relative to the PAM (blue) indicated by the numbers underneath. Values and error bars represent the mean and standard deviation of three independent biological replicates.

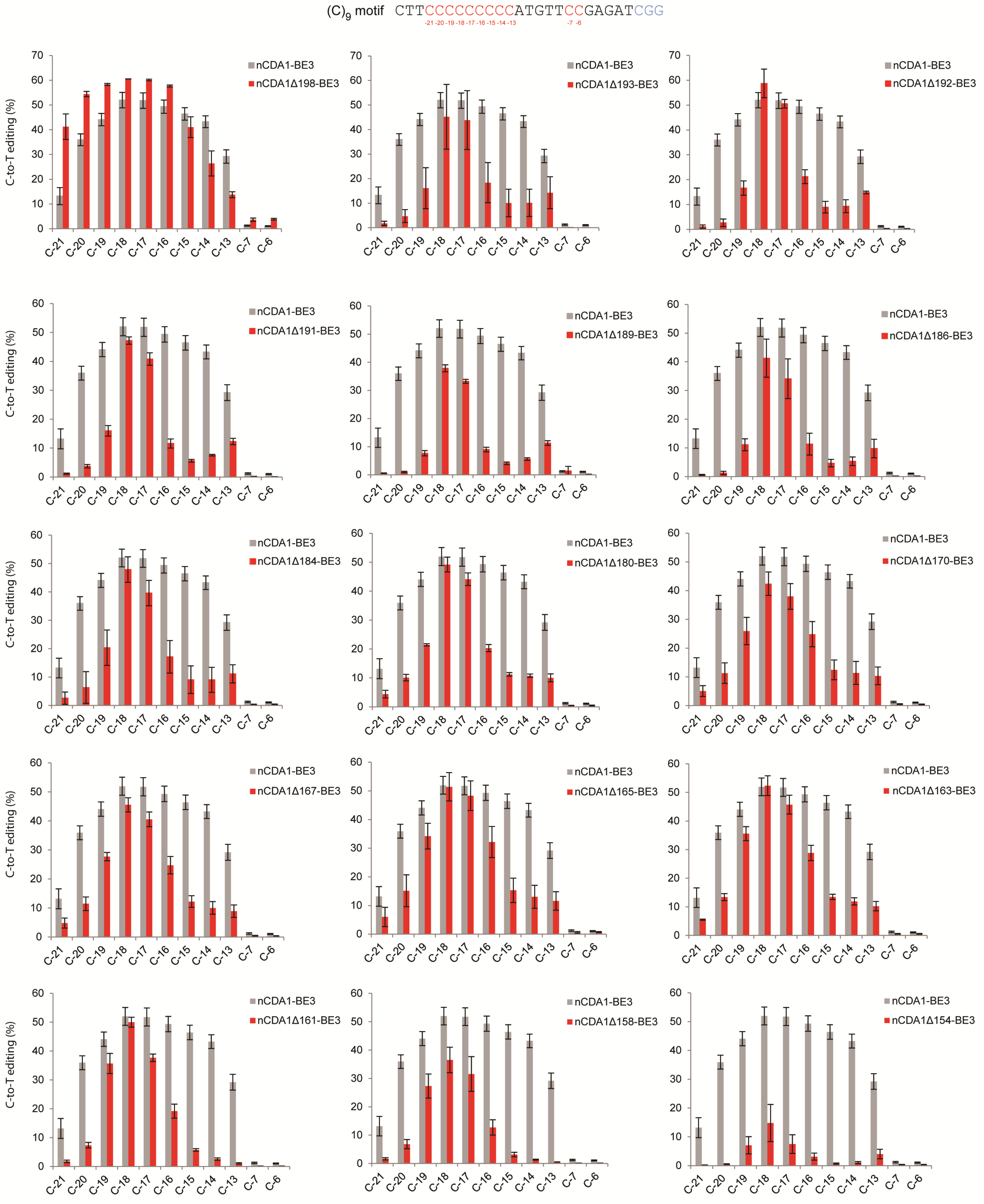

Supplementary Figure 10 Effects of C-terminal truncations of the CDA1 domain on the width of the editing window of nCDA1-BE3 base editors in a (C)9 motif. Cs in the target region are shown in red, with the number below indicating their distance from the PAM (blue). The C-to-T conversion efficiencies are plotted for all Cs within the protospacer, and

shown in comparison to the nCDA1-BE3 base editor with the full-length CDA1 (grey bars). Values and error bars represent the mean and standard deviation of three biological replicates. For completeness, the selected variants shown in Fig. 4 are also included.

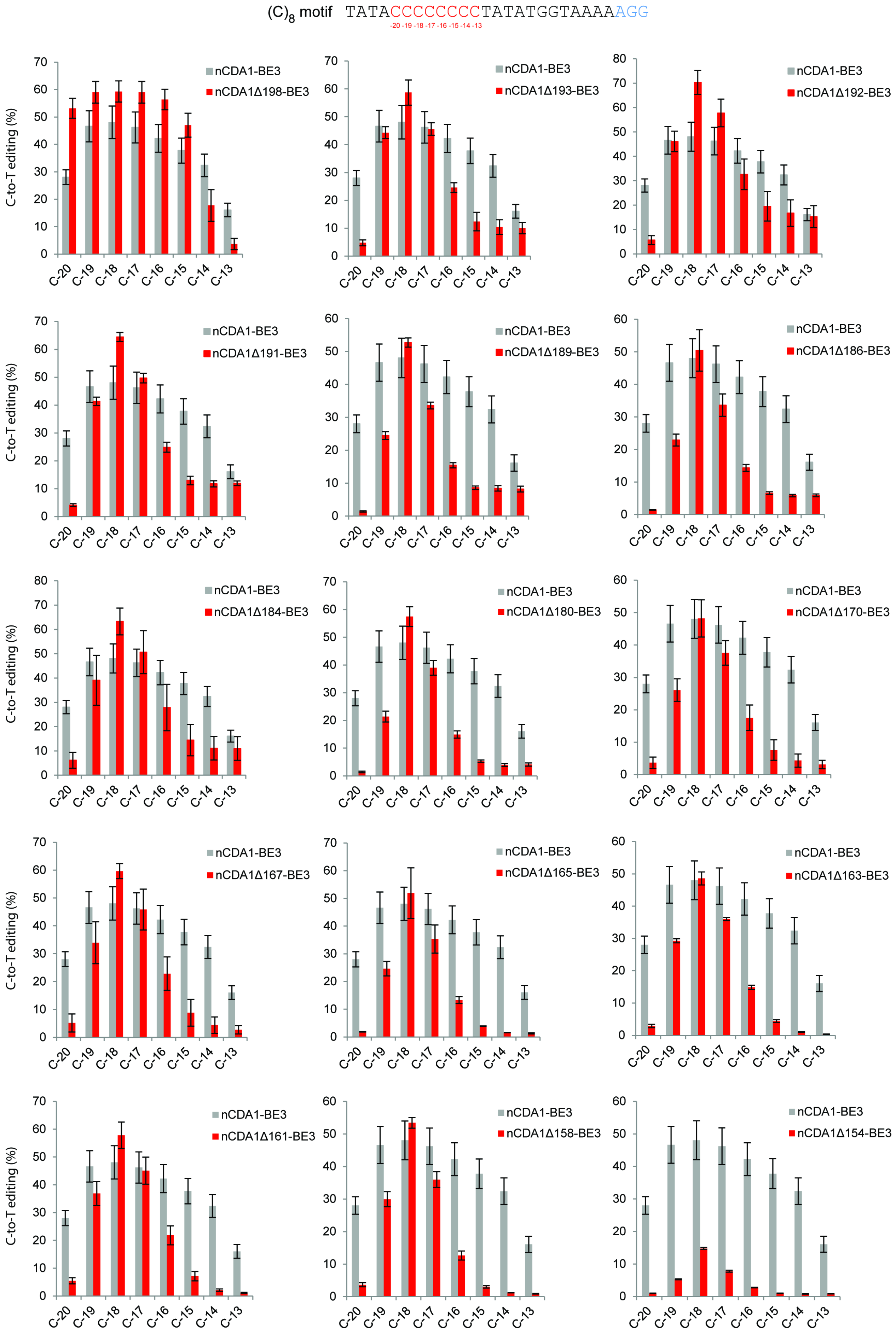
(C)8 motif TATACCCCCCCCTATATGGTAAAAAGG
-20 -19 -18 -17 -16 -15 -14 -13
C-to-T editing (%)
C-20 C-19 C-18 C-17 C-16 C-15 C-14 C-13
nCDA1-BE3
nCDA1Δ198-BE3
nCDA1Δ193-BE3
nCDA1Δ192-BE3
nCDA1Δ191-BE3
nCDA1Δ189-BE3
nCDA1Δ186-BE3
nCDA1Δ184-BE3
nCDA1Δ180-BE3
nCDA1Δ170-BE3
nCDA1Δ167-BE3
nCDA1Δ165-BE3
nCDA1Δ163-BE3
nCDA1Δ161-BE3
nCDA1Δ158-BE3
nCDA1Δ154-BE3

Supplementary Figure 11 Effects of C-terminal truncations of the CDA1 domain on the width of the editing window of nCDA1-BE3 base editors in a $(C)_8$ motif. Cs in the target region are shown in red, with the number below indicating their distance from the PAM (blue). The C-to-T conversion efficiencies are plotted for all Cs within the protospacer, and shown in comparison to the nCDA1-BE3 base editor with the full-length CDA1 (grey bars). Values and error bars represent the mean and standard deviation of three biological replicates. For completeness, the selected variants shown in Fig. 4 are also included.

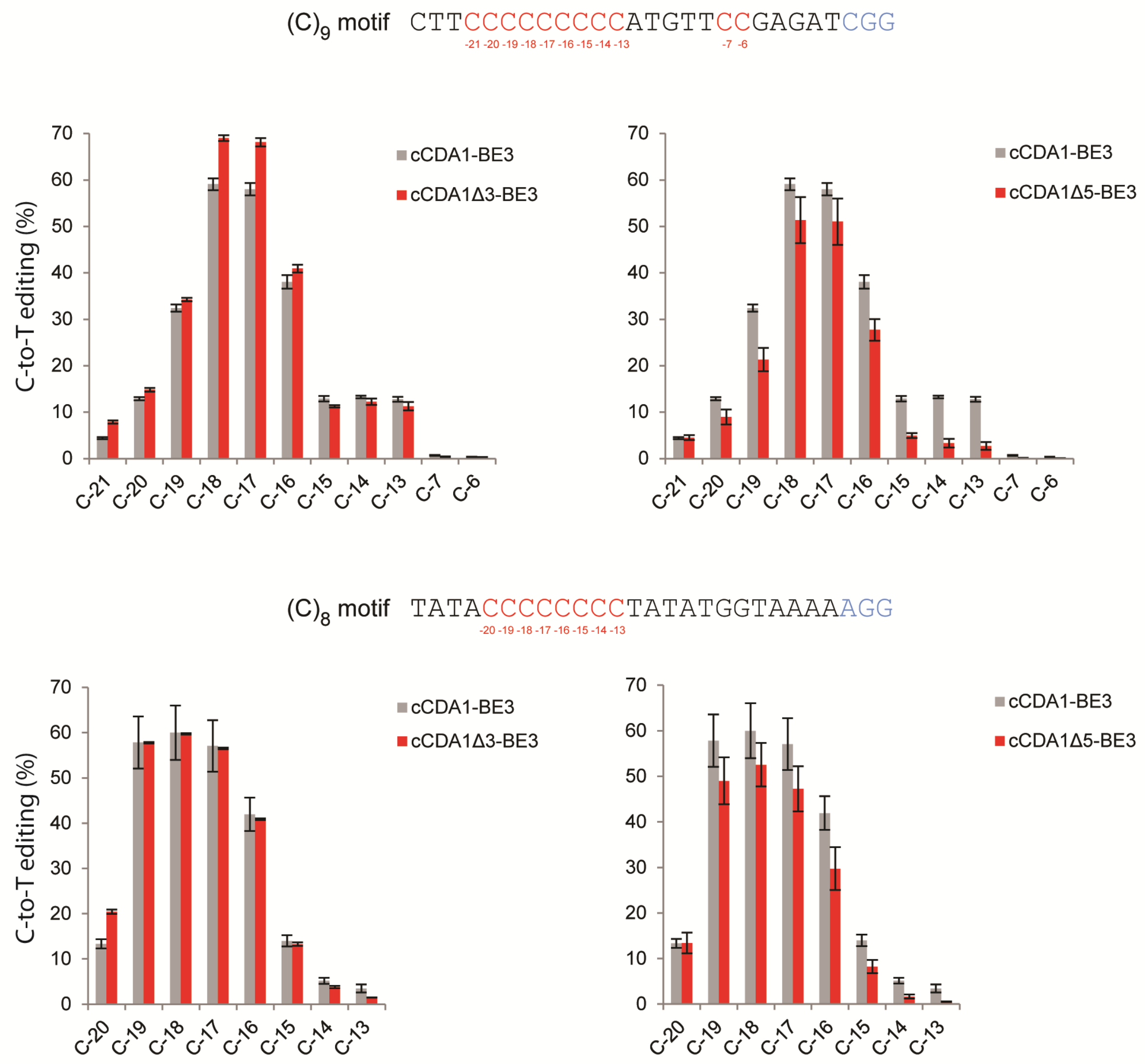

Supplementary Figure 12 Effect of N-terminal CDA1 truncations on the width of the editing window of cCDA1-BE3. The base editor variants were named after the last CDA1 residue deleted. Cs in the target region are shown in red, with the number below indicating their distance from the PAM (blue). The C-to-T conversion efficiencies are plotted for all Cs within the protospacer, and shown in comparison to the cCDA1-BE3 base editor with the full-length CDA1 (grey bars). Values and error bars represent the mean and standard deviation of three biological replicates.

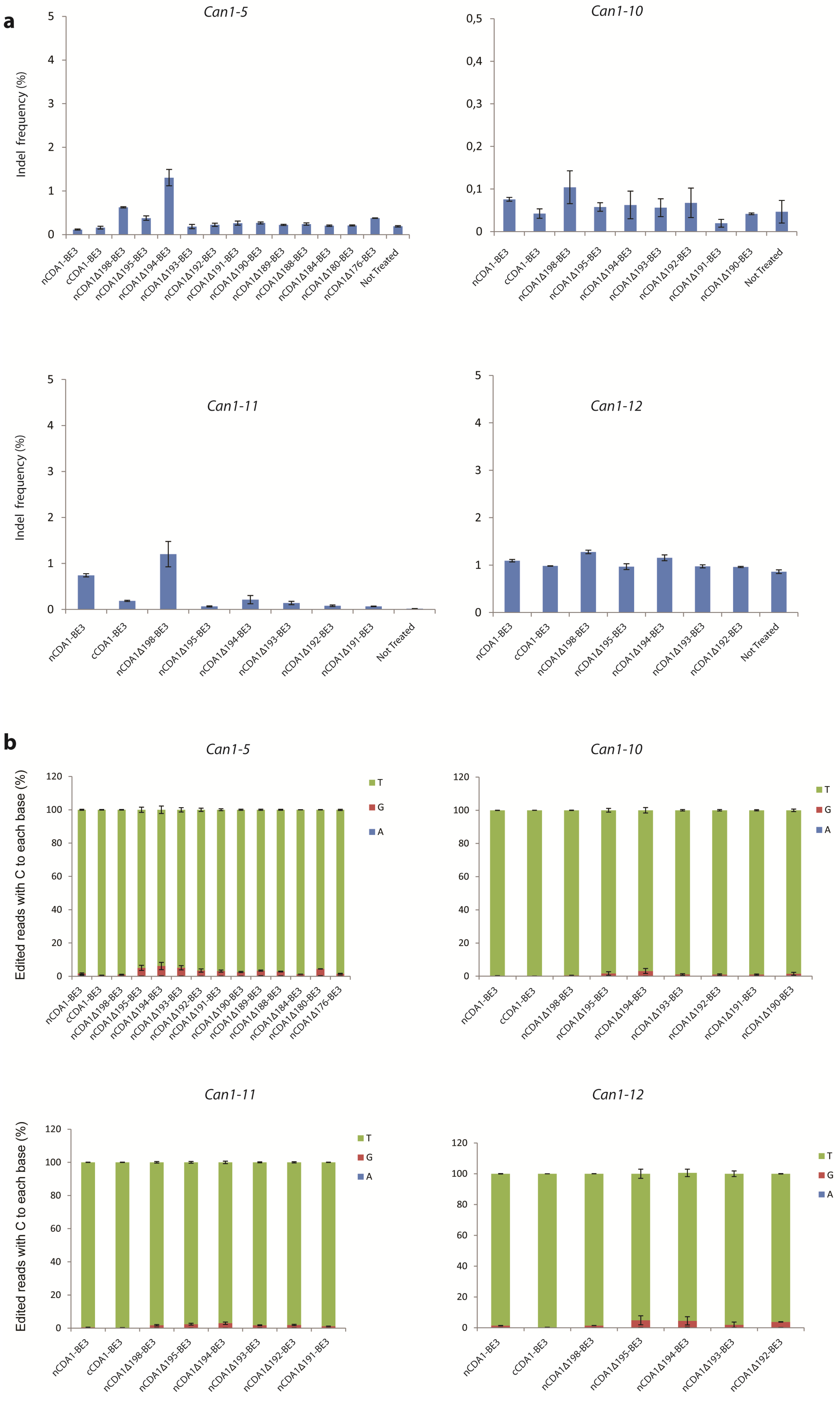
a
Can1-5
Indel frequency (%)
nCDA1-BE3
cCDA1-BE3
nCDA1Δ198-BE3
nCDA1Δ195-BE3
nCDA1Δ194-BE3
nCDA1Δ193-BE3
nCDA1Δ192-BE3
nCDA1Δ191-BE3
nCDA1Δ190-BE3
nCDA1Δ189-BE3
nCDA1Δ188-BE3
nCDA1Δ184-BE3
nCDA1Δ180-BE3
nCDA1Δ176-BE3
Not Treated
Can1-10
Can1-11
Can1-12
b
Edited reads with C to each base (%)
T
G
A

Supplementary Figure 13 Comparison of indel formation frequencies and product purities for nCDA1-BE3, cCDA1-BE3 and the base editors with CDA1 truncations on endogenous yeast gene target sites. **a** Graph showing indel mutation frequencies for nCDA1-BE3, cCDA1-BE3 and the different narrowed-window nCDA1-BE3 variants for four endogenous sites from Fig. 5. **b** Graph showing frequencies of each C_{-18} edited to T, G, or A at the same four sites shown in **(a)**. Values and error bars represent the mean and standard deviation of three biological replicates

Supplementary Table 1 Approximate width of the editing window of base editors with truncated CDA1 domains.

Base editor	$(C)_9$ motif	$(C)_8$ motif
nCDA1-BE3	8	7
nCDA1Δ198-BE3	7	6
nCDA1Δ195-BE3	3	3
nCDA1Δ194-BE3	3	3
nCDA1Δ193-BE3	2	3
nCDA1Δ192-BE3	2	3
nCDA1Δ191-BE3	2	3
nCDA1Δ190-BE3	2	3
nCDA1Δ189-BE3	2	2
nCDA1Δ188-BE3	2	2
nCDA1Δ186-BE3	2	2
nCDA1Δ184-BE3	2	3
nCDA1Δ182-BE3	2	2
nCDA1Δ180-BE3	2	2
nCDA1Δ176-BE3	2	2
nCDA1Δ170-BE3	4	3
nCDA1Δ167-BE3	4	3
nCDA1Δ165-BE3	4	2
nCDA1Δ163-BE3	4	3
nCDA1Δ161-BE3	3	3
nCDA1Δ158-BE3	3	3
nCDA1Δ154-BE3	2	2

The width of the editing window is defined as the number of nucleotides within which editing efficiency exceeds the half-maximal value[23].

Supplementary Table 2 Effect of the induction time on base editing precision.

	cCDA1-BE3			nCDA1Δ190-BE3			nCDA1Δ184-BE3		
	20 h	40 h	60 h	20 h	40 h	60 h	20 h	40 h	60 h
$C_{-19}T_{-18}$ Homozygous	6/12	6/12	4/12	12/12	12/12	12/12	11/12	11/12	10/12
$C_{-19}T_{-18}$ / $T_{-19}T_{-18}$ Heterozygous	5/12	4/12	7/12	0/12	0/12	0/12	0/12	1/12	2/12
$T_{-19}T_{-18}$ Homozygous	1/12	2/12	1/12	0/12	0/12	0/12	1/12	0/12	0/12

Yeast cells were transformed with plasmids expressing each of the three indicated base editors and a sgRNA targeting the same site (*Can1-5*) as shown in Fig. 6, followed by induction of 20, 40 and 60 h. For each base editor, 12 canavanine-resistant colonies were randomly picked from the selection plate followed by sequencing of the *Can1* locus. The different types of edited products are listed in the first column, and the colony numbers representing each product type are given.

Supplementary Table 3 Primers and synthetic nucleotide sequences used in this study.

Generation of sgRNA plasmids

Primer name	Sequence (5' → 3')
sgRNA-Can1-1	AAAGATAAATGATCGATACTAATCCATGCCGCCAGGTTTTAGAGCTAGAAATAGCAAGT
sgRNA-Can1-2	AAAGATAAATGATCGGCAAATTCAAATATTTACGTGTTTTAGAGCTAGAAATAGCAAGT
sgRNA-Can1-3	AAAGATAAATGATCGACGTCCAAAATTGAATGACTGTTTTAGAGCTAGAAATAGCAAGT
sgRNA-Can1-4	AAAGATAAATGATCGTTTCAAGGTACTGAACTAGTGTTTTAGAGCTAGAAATAGCAAGT
sgRNA-Can1-5	AAAGATAAATGATCGTCCAATAACGGAATCCAACTGTTTTAGAGCTAGAAATAGCAAGT
sgRNA-Can1-6	AAAGATAAATGATCGTTCTCAATAGCAATAATAAGTTTTAGAGCTAGAAATAGCAAGT
sgRNA-Can1-7	AAAGATAAATGATCGAAACCAATACATGTAACCATGTTTTAGAGCTAGAAATAGCAAGT
sgRNA-Can1-8	AAAGATAAATGATCGTTGTTCCCTGTCAAATATTAGTTTTAGAGCTAGAAATAGCAAGT
sgRNA-Can1-9	AAAGATAAATGATCGCTAAGGATAAAAACGAAGGGGTTTTAGAGCTAGAAATAGCAAGT
sgRNA-Can1-10	AAAGATAAATGATCGCCCTGGAACTTAGTGTAGTGTTTTAGAGCTAGAAATAGCAAGT
sgRNA-Can1-11	AAAGATAAATGATCGTTCTCTATGGAGGATGGCATGTTTTAGAGCTAGAAATAGCAAGT
sgRNA-Can1-12	AAAGATAAATGATCGTGCCTCAATGTCTCTTCTATGTTTTAGAGCTAGAAATAGCAAGT
sgRNA-C_9 motif	AAAGATAAATGATCGCCCCCCCCCATGTTCCGAGATGTTTTAGAGCTAGAAATAGCAAGT
sgRNA-C_{19} motif	AAAGATAAATGATCGCCCCCCCCCCCCGCATCAACGTTTTAGAGCTAGAAATAGCAAGT
sgRNA-C_8 motif	AAAGATAAATGATCGCCCCCCCCTATATGGTAAAAGTTTTAGAGCTAGAAATAGCAAGT
sgRNA-Rev	TATAGGGCGAATTGGGTACCGGCCGCAAATTAAAG

Generation of base editors with different linker lengths

Primer name	Sequence (5' → 3')	Experiment
BE3-rApobec1-1F	AGAAAAAACCCCGGATTCTAGAACTAGTGGATCCCCCGGGAAAAAAATGAGTTCCGAGACAGGC	Introduction of rAPOBEC1
BE3-rApobec1-1R	CAGATTCAGAAGTACCTGGAGTTTCAGAACCAGATTTCAACCCTGTGGCCCA	
BE3-rApobec1-2F	TCCAGGTACTTCTGAATCTGCTACTCCAGAATCTATGGACAAGAAGTACTCC	
BE3-rApobec1-2R	ATTACTAAAGATCTCCTGCAGGTAGCAGATCCGATTCTTTC	

BE3-D10A-F	ACTCCATTGGGCTCGCTATCGGCACAAACAGC	D10A point mutation
BE3-D10A-R	GCTGTTTGTGCCGATAGCGAGCCCAATGGAGT	
BE3-UGI-1F	ACTTGTTTACTCTGACCAACTTGGGCGCGCCTGCAGCCTTCAA	Introduction of UGI
BE3-UGI-1R	TTGAACCACCAGAGTCTCCACCGAGCTGAGAGAGG	
BE3-UGI-2F	GCTCGGTGGAGACTCTGGTGGTTCAACTAATTTATC	
BE3-UGI-2R	TCCTCTTCTTCTTGGGTGAACCACCAGACAACAT	
BE3-UGI-3F	GTCTGGTGGTTCACCCAAGAAGAAGAGGAAGG	
BE3-UGI-3R	TTCAGTATAATGTTACATGCGTACACGCGTCTGTACAGAAAAAAAAGAAAAAATTTG	
BE-NL-1R	TCTTGTCCATTTTCAACCCTGTGGCCCA	BE-NL
BE-NL-2F	TCTTGTCCATTGGAGCTGGAGCTGGTTTCAACCCTGTGGCCCA	
BE-PAP-1R	TTGTCCATTGGAGCTGGTTTCAACCCTGTGGCCCA	BE-PAP
BE-PAP-2F	AAACCAGCTCCAATGGACAAGAAGTACTCCATTGGGCTCGCT	
BE-PAPAP-1R	TCTTGTCCATTGGAGCTGGAGCTGGTTTCAACCCTGTGGCCCA	BE-PAPAP
BE-PAPAP-2F	TCCAGCTCCAATGGACAAGAAGTACTCCATTGGGCTCGCT	
BE-PAPAPA-1R	TGGCTGGTGCTGGAGCTGGTTTCAACCCTGTGGCCCACAG	BE-PAPAPA
BE-PAPAPA-2F	ACCAGCTCCAGCACCAGCCATGGACAAGAAGTACTCCAT	
BE-PAPAPAP-1R	AGGGGCAGGGGCTGGTGCTGGAGCTGGTTTCAACCCTGTGGCCCACAG	BE-PAPAPAP
BE-PAPAPAP-2F	CAGCTCCAGCACCAGCCCCTATGGACAAGAAGTACTCCAT	
BE-PAPAPAPA-1R	GGCAGGGGCTGGTGCTGGAGCTGGTTTCAACCCTGTGGCCCACAG	BE-PAPAPAPA
BE-PAPAPAPA-2F	CTCCAGCACCAGCCCCTGCCATGGACAAGAAGTACTCCAT	
BE-P(AP)4-1R	AGGGGCAGGGGCTGGTGCTGGAGCTGGTTTCAACCCTGTGGCCCACAG	BE-P(AP)4
BE-P(AP)4-2F	CAGCACCAGCCCCTGCCCCTATGGACAAGAAGTACTCCAT	
BE-P(AP)7-1R	AGCAGGTGCAGGTGCTGGTGCGGGTGCAGGTTTCAACCCTGTGGCCCACAG	BE-P(AP)7
BE-P(AP)7-2F	CACCAGCACCTGCACCTGCTCCAGCTCCCGCTCCAATGGACAAGAAGTACTCCAT	
BE-P(AP)10-1R	CTGGTGCTGGCGCAGGAGCTGGAGCAGGTGCGGGAGCAGGTTTCAACCCTGTGGCCCACAG	BE-P(AP)10
BE-P(AP)10-2F	AGCTCCTGCGCCAGCACCAGCCCCAGCACCCGCACCGGCTCCTATGGACAAGAAGTACTCCAT	
BE-A(EAAAK)2A-1R	CAGCAGCTTCCTTGGCTGCTGCTTCAGCTTTCAACCCTGTGGCCCACAG	BE-A(EAAAK)2A
BE-A(EAAAK)2A-2F	AGCAGCCAAGGAAGCTGCTGCAAAAGCAATGGACAAGAAGTACTCCAT	
BE-A(EAAAK)3A-1R	TCTTTTGCTGCAGCTTCCTTAGCTGCGGCTTCAGCTTTCAACCCTGTGGCCCACAG	BE-A(EAAAK)3A
BE-A(EAAAK)3A-2F	AAGGAAGCTGCAGCAAAAGAAGCCGCTGCAAAAGCCATGGACAAGAAGTACTCCAT	

Generation of cBE3, n/cCDA1-BE3 and n/cCDA1Δ-BE3

Primer name	Sequence (5' → 3')	Experiment
nCDA1-BE3-1F	AGAAAAAACCCCGGATTCTAGAACTAGTGGATCCCCCGGG AAAAAAATGACCGACGCTGAGTACGTGAG	nCDA1-BE3
nCDA1-BE3-1R	CAGATTCAGAAGTACCTGGAGTTTCAGAACCAGAAACAGC AGGACTCTTAGTGGTGT	
cCDA1-BE3-UGI-F	TCCTGCTGTTTCTAGATCTGGTGGTTCAACTAATTTATC	Introduction of UGI
cCDA1-BE3-UGI-R	ATCCGGAGCCTCTAGATCACACCTTCCTCTTCTTCTTGGG G	
cCDA1-BE3-XTEN-1F	CTCTGACCAACTTGGGCGCGCCTGCAGCCTTCAAGTACTT	Introduction of XTEN linker
cCDA1-BE3-XTEN-1R	CAGATTCAGAAGTACCTGGAGTTTCAGAACCAGAGTCTCC ACCGAGCTGAGAGA	
cCDA1-BE3-XTEN-2F	TCCAGGTACTTCTGAATCTGCTACTCCAGAATCTATGACC GACGCTGAGTACGTGAG	
cCDA1-BE3-XTEN-2R	ATTTGCAGGCATTTGCTCGGCATGCCGGTAGAGGTGTGG T	
cBE3-2F	TCCAGGTACTTCTGAATCTGCTACTCCAGAATCTATGAGTT CCGAGACAGGCCC	cBE3
cBE3-2R	AACCACCAGATTTCAACCCTGTGGCCCACA	
cBE3-3F	AGGGTTGAAATCTGGTGGTTCAACTAATTT	
CDA1-Cas-1F	AGAAAAAACCCCGGATTCTAGAACTAGTGGATCCCCCGGG AAAAAAATGACCGACGCTGAGTACGTGAG	nCDA1-NL-BE3
CDA1-Cas-1R	TCTTGTCCATAACAGCAGGACTCTTAGTGG	
CDA1-Cas-2F	TCCTGCTGTTATGGACAAGAAGTACTCCAT	
CDA1-Cas-2R	ATTACTAAAGATCTCCTGCAGGTAGCAGATCCGATTCTTTC	
Cas- CDA1-1F	CTCTGACCAACTTGGGCGCGCCTGCAGCCTTCAAGTACTT	cCDA1-NL-BE3
Cas- CDA1-1R	CGTCGGTCATGTCTCCACCGAGCTGAGAGA	
Cas- CDA1-2F	CGGTGGAGACATGACCGACGCTGAGTACGTGAG	
Cas- CDA1-2R	ATTTGCAGGCATTTGCTCGGCATGCCGGTAGAGGTGTGG T	
198CDA1-Cas-1R	TCTTGTCCATTTTTACCTGAATCATAATGG	nCDA1Δ198-BE3
198CDA1-Cas-2F	TCAGGTAAAAATGGACAAGAAGTACTCCAT	
195CDA1-Cas-1R	TCTTGTCCATAATCATAATGGACAACTCGCTC	nCDA1Δ195-BE3
195CDA1-Cas-2F	CATTATGATTATGGACAAGAAGTACTCCAT	
194CDA1-Cas-1R	TCTTGTCCATCATAATGGACAACTCGCTC	nCDA1Δ194-BE3
194CDA1-Cas-2F	GTCCATTATGATGGACAAGAAGTACTCCAT	
193CDA1-Cas-1R	TCTTGTCCATAATGGACAACTCGCTCCGTCG	nCDA1Δ193-BE3
193CDA1-Cas-2F	GTTGTCCATTATGGACAAGAAGTACTCCAT	
192CDA1-Cas-1R	TCTTGTCCATGGACAACTCGCTCCGTCGTTTT	nCDA1Δ192-BE3
192CDA1-Cas-2F	CGAGTTGTCCATGGACAAGAAGTACTCCAT	
191CDA1-Cas-1R	TCTTGTCCATCAACTCGCTCCGTCGTTTTTCA	nCDA1Δ191-BE3
191CDA1-Cas-2F	GAGCGAGTTGATGGACAAGAAGTACTCCAT	
190CDA1-Cas-1R	TCTTGTCCATCTCGCTCCGTCGTTTTTCAGC	nCDA1Δ190-BE3
190CDA1-Cas-2F	ACGGAGCGAGATGGACAAGAAGTACTCCAT	
189CDA1-Cas-1R	TCTTGTCCATGCTCCGTCGTTTTTCAGCTC	nCDA1Δ189-BE3

189CDA1-Cas-2F	ACGACGGAGCATGGACAAGAAGTACTCCAT	
188CDA1-Cas-1R	TCTTGTCCATCCGTCGTTTTTCAGCTCGCT	nCDA1Δ188-BE3
188CDA1-Cas-2F	AAAACGACGGATGGACAAGAAGTACTCCAT	
186CDA1-Cas-1R	TCTTGTCCATTTTTTCAGCTCGCTTCAAAG	nCDA1Δ186-BE3
186CDA1-Cas-2F	AGCTGAAAAAATGGACAAGAAGTACTCCAT	
184CDA1-Cas-1R	TCTTGTCCATAGCTCGCTTCAAAGTCTTCT	nCDA1Δ184-BE3
184CDA1-Cas-2F	GAAGCGAGCTATGGACAAGAAGTACTCCAT	
182CDA1-Cas-1R	TCTTGTCCATCTTCAAAGTCTTCTCAAGCC	nCDA1Δ182-BE3
182CDA1-Cas-2F	GACTTTGAAGATGGACAAGAAGTACTCCAT	
180CDA1-Cas-1R	TCTTGTCCATAGTCTTCTCAAGCCATCTAT	nCDA1Δ180-BE3
180CDA1-Cas-2F	TGAGAAGACTATGGACAAGAAGTACTCCAT	
176CDA1-Cas-1R	TCTTGTCCATCCATCTATTCTCATTCAATT	nCDA1Δ176-BE3
176CDA1-Cas-2F	GAATAGATGGATGGACAAGAAGTACTCCAT	
170CDA1-Cas-1R	TCTTGTCCATTTGATTGTGCGACGATTGGA	nCDA1Δ170-BE3
170CDA1-Cas-2F	GCACAATCAAATGGACAAGAAGTACTCCAT	
167CDA1-Cas-1R	TCTTGTCCATCGACGATTGGATGAATATTT	nCDA1Δ167-BE3
167CDA1-Cas-2F	CCAATCGTCGATGGACAAGAAGTACTCCAT	
165CDA1-Cas-1R	TCTTGTCCATTTGGATGAATATTTTCCTGC	nCDA1Δ165-BE3
165CDA1-Cas-2F	ATTCATCCAAATGGACAAGAAGTACTCCAT	
163CDA1-Cas-1R	TCTTGTCCATGAATATTTTCCTGCAACATT	nCDA1Δ163-BE3
163CDA1-Cas-2F	GAAAATATTCATGGACAAGAAGTACTCCAT	
161CDA1-Cas-1R	TCTTGTCCATTTTCCTGCAACATTGGTAGT	nCDA1Δ161-BE3
161CDA1-Cas-2F	TTGCAGGAAAATGGACAAGAAGTACTCCAT	
158CDA1-Cas-1R	TCTTGTCCATACATTGGTAGTGTTCACTTA	nCDA1Δ158-BE3
158CDA1-Cas-2F	CTACCAATGTATGGACAAGAAGTACTCCAT	
154CDA1-Cas-1R	TCTTGTCCATTTCACTTACCATTACATTCA	nCDA1Δ154-BE3
154CDA1-Cas-2F	GGTAAGTGAAATGGACAAGAAGTACTCCAT	
Cas-4-208CDA1-1R	CGTACTCAGCGTCTCCACCGAGCTGAGAGA	cCDA1Δ3-BE3
Cas-4-208CDA1-2F	CGGTGGAGACGCTGAGTACGTGAGAATCCA	
Cas-6-208CDA1-1R	TTCTCACGTAGTCTCCACCGAGCTGAGAGA	cCDA1Δ5-BE3
Cas-6-208CDA1-2F	CGGTGGAGACTACGTGAGAATCCATGAGAA	

Primers to amplify target regions for NGS

**Primer name**	**Sequence (5' → 3')**
C9 motif-NGS-index1-F	CGATGTACTGCGGAAGTGAGGGGAGC
C9 motif-NGS-index1-R	TGGTCATATCCGTGCGCGTAATCCTTCT
C9 motif-NGS-index2-F	ATCACGACTGCGGAAGTGAGGGGAGC
C9 motif-NGS-index2-R	GCCTAATATCCGTGCGCGTAATCCTTCT
C9 motif-NGS-index3-F	AGTTCCACTGCGGAAGTGAGGGGAGC
C9 motif-NGS-index3-R	CTCTACTATCCGTGCGCGTAATCCTTCT
C9 motif-NGS-index4-F	CACTCAACTGCGGAAGTGAGGGGAGC

C9 motif-NGS-index4-R	TGTTGGTATCCGTGCGCGTAATCCTTCT
C9 motif-NGS-index5-F	GTGGCCACTGCGGAAGTGAGGGGAGC
C9 motif-NGS-index5-R	CGAAACTATCCGTGCGCGTAATCCTTCT
C9 motif-NGS-index6-F	CGTACGACTGCGGAAGTGAGGGGAGC
C9 motif-NGS-index6-R	CCACTCTATCCGTGCGCGTAATCCTTCT
C9 motif-NGS-index7-F	GGTAGCACTGCGGAAGTGAGGGGAGC
C9 motif-NGS-index7-R	ATCAGTTATCCGTGCGCGTAATCCTTCT
C9 motif-NGS-index8-F	CACCGGACTGCGGAAGTGAGGGGAGC
C9 motif-NGS-index8-R	ATCGTGTATCCGTGCGCGTAATCCTTCT
C9 motif-NGS-index9-F	ATGAGCACTGCGGAAGTGAGGGGAGC
C9 motif-NGS-index9-R	AGGAATTATCCGTGCGCGTAATCCTTCT
C9 motif-NGS-index10-F	CAAAAGACTGCGGAAGTGAGGGGAGC
C9 motif-NGS-index10-R	TAGTTGTATCCGTGCGCGTAATCCTTCT
C9 motif-NGS-index11-F	TCGGCAACTGCGGAAGTGAGGGGAGC
C9 motif-NGS-index11-R	GAATGATATCCGTGCGCGTAATCCTTCT
C9 motif-NGS-index12-F	TCCCGAACTGCGGAAGTGAGGGGAGC
C9 motif-NGS-index12-R	CTTCGATATCCGTGCGCGTAATCCTTCT
C9 motif-NGS-index13-F	CTATACACTGCGGAAGTGAGGGGAGC
C9 motif-NGS-index13-R	TCTGAGTATCCGTGCGCGTAATCCTTCT
C9 motif-NGS-index14-F	TTAGGCACTGCGGAAGTGAGGGGAGC
C9 motif-NGS-index14-R	TGACCATATCCGTGCGCGTAATCCTTCT
C9 motif-NGS-index15-F	ACATGTACTGCGGAAGTGAGGGGAGC
C9 motif-NGS-index15-F	CAGATCTATCCGTGCGCGTAATCCTTCT
C9 motif-NGS-index16-F	ACTTGAACTGCGGAAGTGAGGGGAGC
C9 motif-NGS-index16-R	GATCAGTATCCGTGCGCGTAATCCTTCT
C9 motif-NGS-index17-F	TAGCTTACTGCGGAAGTGAGGGGAGC
C9 motif-NGS-index17-R	GGCTACTATCCGTGCGCGTAATCCTTCT
C9 motif-NGS-index18-F	CCGTCCACTGCGGAAGTGAGGGGAGC
C9 motif-NGS-index18-R	GTAGAGTATCCGTGCGCGTAATCCTTCT
C9 motif-NGS-index19-F	GTCCGCACTGCGGAAGTGAGGGGAGC
C9 motif-NGS-index19-R	GTGAAATATCCGTGCGCGTAATCCTTCT
C9 motif-NGS-index20-F	GTTTCGACTGCGGAAGTGAGGGGAGC
C9 motif-NGS-index20-R	GAGTGGTATCCGTGCGCGTAATCCTTCT
C9 motif-NGS-index21-F	ACTGATACTGCGGAAGTGAGGGGAGC
C9 motif-NGS-index21-R	ATTCCTTATCCGTGCGCGTAATCCTTCT
C9 motif-NGS-index22-F	CAACTAACTGCGGAAGTGAGGGGAGC
C9 motif-NGS-index22-R	CACGATTATCCGTGCGCGTAATCCTTCT
C9 motif-NGS-index23-F	CAGGCGACTGCGGAAGTGAGGGGAGC
C9 motif-NGS-index23-R	CATGGCTATCCGTGCGCGTAATCCTTCT
C9 motif-NGS-index24-F	ACAGTGACTGCGGAAGTGAGGGGAGC
C9 motif-NGS-index24-R	GCCAATTATCCGTGCGCGTAATCCTTCT

C9 motif-NGS-index25-F	CTTGTAACTGCGGAAGTGAGGGGAGC
C9 motif-NGS-index25-R	AGTCAATATCCGTGCGCGTAATCCTTCT
C9 motif-NGS-index26-F	ATGTCAACTGCGGAAGTGAGGGGAGC
C9 motif-NGS-index26-R	CATTTTTATCCGTGCGCGTAATCCTTCT
C9 motif-NGS-index27-F	CCAACAACTGCGGAAGTGAGGGGAGC
C9 motif-NGS-index27-R	CGGAATTATCCGTGCGCGTAATCCTTCT
C9 motif-NGS-index28-F	TCATTCACTGCGGAAGTGAGGGGAGC
C9 motif-NGS-index28-R	CTAGCTTATCCGTGCGCGTAATCCTTCT
C9 motif-NGS-index29-F	TCGAAGACTGCGGAAGTGAGGGGAGC
C9 motif-NGS-index29-R	CTCAGATATCCGTGCGCGTAATCCTTCT
C9 motif-NGS-index30-F	GACGACACTGCGGAAGTGAGGGGAGC
C9 motif-NGS-index30-R	TAATCGTATCCGTGCGCGTAATCCTTCT
C9 motif-NGS-index31-F	TACAGCACTGCGGAAGTGAGGGGAGC
C9 motif-NGS-index31-R	ATAAGTTATCCGTGCGCGTAATCCTTCT
C19 motif-NGS-index1-F	AGTCAAATCTTCCATATACCCTGGCTCT
C19 motif-NGS-index1-R	ATGTCAGCTACGACATCATCTACAGGAAG
C8 motif-NGS-index1-F	ATCACGTTCATCTATAAGGATATGGGTCG
C8 motif-NGS-index1-R	CGATGTATAGCATATAATAAAAGTGGAG
C8 motif-NGS-index2-F	TTAGGCTTCATCTATAAGGATATGGGTCG
C8 motif-NGS-index2-R	TGACCAATAGCATATAATAAAAGTGGAG
C8 motif-NGS-index3-F	ACAGTGTTCATCTATAAGGATATGGGTCG
C8 motif-NGS-index3-R	GCCAATATAGCATATAATAAAAGTGGAG
C8 motif-NGS-index4-F	CAGATCTTCATCTATAAGGATATGGGTCG
C8 motif-NGS-index4-R	ACTTGAATAGCATATAATAAAAGTGGAG
C8 motif-NGS-index5-F	GATCAGTTCATCTATAAGGATATGGGTCG
C8 motif-NGS-index5-R	TAGCTTATAGCATATAATAAAAGTGGAG
C8 motif-NGS-index6-F	GGCTACTTCATCTATAAGGATATGGGTCG
C8 motif-NGS-index6-R	CTTGTAATAGCATATAATAAAAGTGGAG
C8 motif-NGS-index7-F	AGTCAATTCATCTATAAGGATATGGGTCG
C8 motif-NGS-index7-R	AGTTCCATAGCATATAATAAAAGTGGAG
C8 motif-NGS-index8-F	ATGTCATTCATCTATAAGGATATGGGTCG
C8 motif-NGS-index8-R	CCGTCCATAGCATATAATAAAAGTGGAG
C8 motif-NGS-index9-F	GTAGAGTTCATCTATAAGGATATGGGTCG
C8 motif-NGS-index9-R	GTCCGCATAGCATATAATAAAAGTGGAG
C8 motif-NGS-index10-F	GTGAAATTCATCTATAAGGATATGGGTCG
C8 motif-NGS-index10-R	GTGGCCATAGCATATAATAAAAGTGGAG
C8 motif-NGS-index11-F	GTTTCGTTCATCTATAAGGATATGGGTCG
C8 motif-NGS-index11-R	CGTACGATAGCATATAATAAAAGTGGAG
C8 motif-NGS-index12-F	GAGTGGTTCATCTATAAGGATATGGGTCG
C8 motif-NGS-index12-R	GGTAGCATAGCATATAATAAAAGTGGAG
C8 motif-NGS-index13-F	ACTGATTTCATCTATAAGGATATGGGTCG

C8 motif-NGS-index13-R	ATGAGCATAGCATATAATAAAAGTGGAG
C8 motif-NGS-index14-F	ATTCCTTTCATCTATAAGGATATGGGTCG
C8 motif-NGS-index14-R	CAAAAGATAGCATATAATAAAAGTGGAG
C8 motif-NGS-index15-F	CAACTATTCATCTATAAGGATATGGGTCG
C8 motif-NGS-index15-R	CACCGGATAGCATATAATAAAAGTGGAG
C8 motif-NGS-index16-F	CACGATTTCATCTATAAGGATATGGGTCG
C8 motif-NGS-index16-R	CACTCAATAGCATATAATAAAAGTGGAG
C8 motif-NGS-index17-F	CAGGCGTTCATCTATAAGGATATGGGTCG
C8 motif-NGS-index17-R	CATGGCATAGCATATAATAAAAGTGGAG
C8 motif-NGS-index18-F	CATTTTTTCATCTATAAGGATATGGGTCG
C8 motif-NGS-index18-R	CCAACAATAGCATATAATAAAAGTGGAG
C8 motif-NGS-index19-F	CGGAATTTCATCTATAAGGATATGGGTCG
C8 motif-NGS-index19-R	CTAGCTATAGCATATAATAAAAGTGGAG
C8 motif-NGS-index20-F	CTATACTTCATCTATAAGGATATGGGTCG
C8 motif-NGS-index20-R	CTCAGAATAGCATATAATAAAAGTGGAG
C8 motif-NGS-index21-F	GACGACTTCATCTATAAGGATATGGGTCG
C8 motif-NGS-index21-R	TAATCGATAGCATATAATAAAAGTGGAG
C8 motif-NGS-index22-F	TACAGCTTCATCTATAAGGATATGGGTCG
C8 motif-NGS-index22-R	TATAATATAGCATATAATAAAAGTGGAG
C8 motif-NGS-index23-F	TCATTCTTCATCTATAAGGATATGGGTCG
C8 motif-NGS-index23-R	TCCCGAATAGCATATAATAAAAGTGGAG
C8 motif-NGS-index24-F	TCGAAGTTCATCTATAAGGATATGGGTCG
C8 motif-NGS-index24-R	TCGGCAATAGCATATAATAAAAGTGGAG
C8 motif-NGS-index25-F	GCCTAATTCATCTATAAGGATATGGGTCG
C8 motif-NGS-index25-R	TGGTCAATAGCATATAATAAAAGTGGAG
C8 motif-NGS-index26-F	CTCTACTTCATCTATAAGGATATGGGTCG
C8 motif-NGS-index26-R	TGTTGGATAGCATATAATAAAAGTGGAG
C8 motif-NGS-index27-F	CGAAACTTCATCTATAAGGATATGGGTCG
C8 motif-NGS-index27-R	CCACTCATAGCATATAATAAAAGTGGAG
C8 motif-NGS-index28-F	ATAAGTTTCATCTATAAGGATATGGGTCG
C8 motif-NGS-index28-R	ATCGTGATAGCATATAATAAAAGTGGAG
Can1-3-NGS-index1-F	ATCACGAGATTCCTTTCTCCAGCATT
Can1-3-NGS-index1-R	GCCTAACTTTGATGGAAGCGACCCAGA
Can1-3-NGS-index2-F	CGATGTAGATTCCTTTCTCCAGCATT
Can1-3-NGS-index2-R	TGGTCACTTTGATGGAAGCGACCCAGA
Can1-3-NGS-index3-F	AGTTCCAGATTCCTTTCTCCAGCATT
Can1-3-NGS-index3-R	CTCTACCTTTGATGGAAGCGACCCAGA
Can1-3-NGS-index4-F	CACTCAAGATTCCTTTCTCCAGCATT
Can1-3-NGS-index4-R	TGTTGGCTTTGATGGAAGCGACCCAGA
Can1-3-NGS-index5-F	GTGGCCAGATTCCTTTCTCCAGCATT
Can1-3-NGS-index5-R	CGAAACCTTTGATGGAAGCGACCCAGA

Can1-3-NGS-index6-F	CGTACGAGATTCCTTTCTCCAGCATT
Can1-3-NGS-index6-R	CCACTCCTTTGATGGAAGCGACCCAGA
Can1-3-NGS-index7-F	GGTAGCAGATTCCTTTCTCCAGCATT
Can1-3-NGS-index7-R	ATCAGTCTTTGATGGAAGCGACCCAGA
Can1-3-NGS-index8-F	CACCGGAGATTCCTTTCTCCAGCATT
Can1-3-NGS-index8-R	ATCGTGCTTTGATGGAAGCGACCCAGA
Can1-5-NGS-index1-F	ATCACGTTGTTCCCTGTCAAATATTA
Can1-5-NGS-index1-R	CGATGTTTCAGTACCTTGAAATGTGA
Can1-5-NGS-index2-F	TTAGGCTTGTTCCCTGTCAAATATTA
Can1-5-NGS-index2-R	TGACCATTCAGTACCTTGAAATGTGA
Can1-5-NGS-index3-F	ACAGTGTTGTTCCCTGTCAAATATTA
Can1-5-NGS-index3-R	GCCAATTTCAGTACCTTGAAATGTGA
Can1-5-NGS-index4-F	CAGATCTTGTTCCCTGTCAAATATTA
Can1-5-NGS-index4-R	ACTTGATTCAGTACCTTGAAATGTGA
Can1-5-NGS-index5-F	GATCAGTTGTTCCCTGTCAAATATTA
Can1-5-NGS-index5-R	TAGCTTTTCAGTACCTTGAAATGTGA
Can1-5-NGS-index6-F	GGCTACTTGTTCCCTGTCAAATATTA
Can1-5-NGS-index6-R	CTTGTATTCAGTACCTTGAAATGTGA
Can1-5-NGS-index7-F	AGTCAATTGTTCCCTGTCAAATATTA
Can1-5-NGS-index7-R	AGTTCCTTCAGTACCTTGAAATGTGA
Can1-5-NGS-index8-F	ATGTCATTGTTCCCTGTCAAATATTA
Can1-5-NGS-index8-R	CCGTCCTTCAGTACCTTGAAATGTGA
Can1-5-NGS-index9-F	GTAGAGTTGTTCCCTGTCAAATATTA
Can1-5-NGS-index9-R	GTCCGCTTCAGTACCTTGAAATGTGA
Can1-5-NGS-index10-F	GTGAAATTGTTCCCTGTCAAATATTA
Can1-5-NGS-index10-R	GTGGCCTTCAGTACCTTGAAATGTGA
Can1-5-NGS-index11-F	GTTTCGTTGTTCCCTGTCAAATATTA
Can1-5-NGS-index11-R	CGTACGTTCAGTACCTTGAAATGTGA
Can1-5-NGS-index12-F	GAGTGGTTGTTCCCTGTCAAATATTA
Can1-5-NGS-index12-R	GGTAGCTTCAGTACCTTGAAATGTGA
Can1-5-NGS-index13-F	GCCTAATTGTTCCCTGTCAAATATTA
Can1-5-NGS-index13-R	TGGTCATTCAGTACCTTGAAATGTGA
Can1-5-NGS-index14-F	CTCTACTTGTTCCCTGTCAAATATTA
Can1-5-NGS-index14-R	TGTTGGTTCAGTACCTTGAAATGTGA
Can1-5-NGS-index15-F	CGAAACTTGTTCCCTGTCAAATATTA
Can1-5-NGS-index15-R	CCACTCTTCAGTACCTTGAAATGTGA
Can1-5-NGS-index16-F	ATAAGTTTGTTCCCTGTCAAATATTA
Can1-5-NGS-index16-R	ATCGTGTTCAGTACCTTGAAATGTGA
Can1-6-NGS-index1-F	CACCGGATTGGCTCTCTATTATTCAT
Can1-6-NGS-index1-R	ATCGTGAATAAAATACGGGAACCAACG
Can1-6-NGS-index2-F	ATGAGCATTGGCTCTCTATTATTCAT

Can1-6-NGS-index2-R	AGGAATAATAAAATACGGGAACCAACG
Can1-6-NGS-index3-F	CAAAAGATTGGCTCTCTATTATTCAT
Can1-6-NGS-index3-R	TAGTTGAATAAAATACGGGAACCAACG
Can1-6-NGS-index4-F	TCGGCAATTGGCTCTCTATTATTCAT
Can1-6-NGS-index4-R	GAATGAAATAAAATACGGGAACCAACG
Can1-6-NGS-index5-F	TCCCGAATTGGCTCTCTATTATTCAT
Can1-6-NGS-index5-R	CTTCGAAATAAAATACGGGAACCAACG
Can1-6-NGS-index6-F	CTATACATTGGCTCTCTATTATTCAT
Can1-6-NGS-index6-R	TCTGAGAATAAAATACGGGAACCAACG
Can1-6-NGS-index7-F	TTAGGCATTGGCTCTCTATTATTCAT
Can1-6-NGS-index7-R	TGACCAAATAAAATACGGGAACCAACG
Can1-7-NGS-index1-F	ACATGTCACGCAGTCCTTGGGTGAAA
Can1-7-NGS-index1-R	CAGATCACGTCCAAAATTGAATGACT
Can1-7-NGS-index2-F	ACTTGACACGCAGTCCTTGGGTGAAA
Can1-7-NGS-index2-R	GATCAGACGTCCAAAATTGAATGACT
Can1-7-NGS-index3-F	TAGCTTCACGCAGTCCTTGGGTGAAA
Can1-7-NGS-index3-R	GGCTACACGTCCAAAATTGAATGACT
Can1-7-NGS-index4-F	AGTCAACACGCAGTCCTTGGGTGAAA
Can1-7-NGS-index4-R	ATGTCAACGTCCAAAATTGAATGACT
Can1-7-NGS-index5-F	CCGTCCCACGCAGTCCTTGGGTGAAA
Can1-7-NGS-index5-R	GTAGAGACGTCCAAAATTGAATGACT
Can1-7-NGS-index6-F	GTCCGCCACGCAGTCCTTGGGTGAAA
Can1-7-NGS-index6-R	GTGAAAACGTCCAAAATTGAATGACT
Can1-7-NGS-index7-F	GTTTCGCACGCAGTCCTTGGGTGAAA
Can1-7-NGS-index7-R	ACTGATACGTCCAAAATTGAATGACT
Can1-8-NGS-index1-R	GCCTAATCCAATAACGGAATCCAACT
Can1-8-NGS-index2-F	CGATGTGCCCTGGAACTTAGTGTAGT
Can1-8-NGS-index2-R	TGGTCATCCAATAACGGAATCCAACT
Can1-8-NGS-index3-F	AGTTCCGCCCTGGAACTTAGTGTAGT
Can1-8-NGS-index3-R	CTCTACTCCAATAACGGAATCCAACT
Can1-8-NGS-index4-F	CACTCAGCCCTGGAACTTAGTGTAGT
Can1-8-NGS-index4-R	TGTTGGTCCAATAACGGAATCCAACT
Can1-8-NGS-index5-F	GTGGCCGCCCTGGAACTTAGTGTAGT
Can1-8-NGS-index5-R	CGAAACTCCAATAACGGAATCCAACT
Can1-8-NGS-index6-F	CGTACGGCCCTGGAACTTAGTGTAGT
Can1-8-NGS-index6-R	CCACTCTCCAATAACGGAATCCAACT
Can1-8-NGS-index7-F	GGTAGCGCCCTGGAACTTAGTGTAGT
Can1-8-NGS-index7-R	ATCAGTTCCAATAACGGAATCCAACT
Can1-10-NGS-index1-F	ACTGATAGATTCCTTTCTCCAGCATT
Can1-10-NGS-index1-R	CACCGTCACCGTAATATTTGACAGGG
Can1-10-NGS-index2-F	ATTCCTAGATTCCTTTCTCCAGCATT

Can1-10-NGS-index2-R	CAAAAGCACCGTAATATTTGACAGGG
Can1-10-NGS-index3-F	CAACTAAGATTCCTTTCTCCAGCATT
Can1-10-NGS-index3-R	CACCGGCACCGTAATATTTGACAGGG
Can1-10-NGS-index4-F	CACGATAGATTCCTTTCTCCAGCATT
Can1-10-NGS-index4-R	CACTCACACCGTAATATTTGACAGGG
Can1-10-NGS-index5-F	CAGGCGAGATTCCTTTCTCCAGCATT
Can1-10-NGS-index5-R	CATGGCCACCGTAATATTTGACAGGG
Can1-10-NGS-index6-F	CATTTTAGATTCCTTTCTCCAGCATT
Can1-10-NGS-index6-R	CCAACACACCGTAATATTTGACAGGG
Can1-10-NGS-index7-F	CGGAATAGATTCCTTTCTCCAGCATT
Can1-10-NGS-index7-R	CTAGCTCACCGTAATATTTGACAGGG
Can1-10-NGS-index8-F	CTATACAGATTCCTTTCTCCAGCATT
Can1-10-NGS-index8-R	CTCAGACACCGTAATATTTGACAGGG
Can1-10-NGS-index9-F	GACGACAGATTCCTTTCTCCAGCATT
Can1-10-NGS-index9-R	TAATCGCACCGTAATATTTGACAGGG
Can1-10-NGS-index10-F	TACAGCAGATTCCTTTCTCCAGCATT
Can1-10-NGS-index10-R	TATAATCACCGTAATATTTGACAGGG
Can1-10-NGS-index11-F	TCATTCAGATTCCTTTCTCCAGCATT
Can1-10-NGS-index11-R	TCCCGACACCGTAATATTTGACAGGG
Can1-10-NGS-index12-F	TCGAAGAGATTCCTTTCTCCAGCATT
Can1-10-NGS-index12-R	TCGGCACACCGTAATATTTGACAGGG
Can1-11-NGS-index1-F	ATCACGTCACAAACACACCACAGACG
Can1-11-NGS-index1-R	CGATGTTACCAATAGTACCACCAAGGGC
Can1-11-NGS-index2-F	TTAGGCTCACAAACACACCACAGACG
Can1-11-NGS-index2-R	TGACCATACCAATAGTACCACCAAGGGC
Can1-11-NGS-index3-F	ACAGTGTCACAAACACACCACAGACG
Can1-11-NGS-index3-R	GCCAATTACCAATAGTACCACCAAGGGC
Can1-11-NGS-index4-F	CAGATCTCACAAACACACCACAGACG
Can1-11-NGS-index4-R	ACTTGATACCAATAGTACCACCAAGGGC
Can1-11-NGS-index5-F	GATCAGTCACAAACACACCACAGACG
Can1-11-NGS-index5-R	TAGCTTTACCAATAGTACCACCAAGGGC
Can1-11-NGS-index6-F	GGCTACTCACAAACACACCACAGACG
Can1-11-NGS-index6-R	CTTGTATACCAATAGTACCACCAAGGGC
Can1-11-NGS-index7-F	AGTCAATCACAAACACACCACAGACG
Can1-11-NGS-index7-R	AGTTCCTACCAATAGTACCACCAAGGGC
Can1-11-NGS-index8-F	ATGTCATCACAAACACACCACAGACG
Can1-11-NGS-index8-R	CCGTCCTACCAATAGTACCACCAAGGGC
Can1-11-NGS-index9-F	GTAGAGTCACAAACACACCACAGACG
Can1-11-NGS-index9-R	GTCCGCTACCAATAGTACCACCAAGGGC
Can1-11-NGS-index10-F	GTGAAATCACAAACACACCACAGACG
Can1-11-NGS-index10-R	GTGGCCTACCAATAGTACCACCAAGGGC

Can1-11-NGS-index11-F	GTTTCGTCACAAACACACCACAGACG
Can1-11-NGS-index11-R	CGTACGTACCAATAGTACCACCAAGGGC
Can1-12-NGS-index1-F	ACTGATGGTGTTAGCTTTGCTGCCGCC
Can1-12-NGS-index1-R	ATGAGCCTATGCTACAACATTCCAAAATTT
Can1-12-NGS-index2-F	ATTCCTGGTGTTAGCTTTGCTGCCGCC
Can1-12-NGS-index2-R	CAAAAGCTATGCTACAACATTCCAAAATTT
Can1-12-NGS-index3-F	CAACTAGGTGTTAGCTTTGCTGCCGCC
Can1-12-NGS-index3-R	CACCGGCTATGCTACAACATTCCAAAATTT
Can1-12-NGS-index4-F	CACGATGGTGTTAGCTTTGCTGCCGCC
Can1-12-NGS-index4-R	CACTCACTATGCTACAACATTCCAAAATTT
Can1-12-NGS-index5-F	CAGGCGGGTGTTAGCTTTGCTGCCGCC
Can1-12-NGS-index5-R	CATGGCCTATGCTACAACATTCCAAAATTT
Can1-12-NGS-index6-F	CATTTTGGTGTTAGCTTTGCTGCCGCC
Can1-12-NGS-index6-R	CCAACACTATGCTACAACATTCCAAAATTT
Can1-12-NGS-index7-F	CGGAATGGTGTTAGCTTTGCTGCCGCC
Can1-12-NGS-index7-R	CTAGCTCTATGCTACAACATTCCAAAATTT
Can1-12-NGS-index8-F	CTATACGGTGTTAGCTTTGCTGCCGCC
Can1-12-NGS-index8-R	CTCAGACTATGCTACAACATTCCAAAATTT
Can1-12-NGS-index9-F	GACGACGGTGTTAGCTTTGCTGCCGCC
Can1-12-NGS-index9-R	TAATCGCTATGCTACAACATTCCAAAATTT
Can1-12-NGS-index10-F	TACAGCGGTGTTAGCTTTGCTGCCGCC
Can1-12-NGS-index10-R	TATAATCTATGCTACAACATTCCAAAATTT

Supplementary Table 4 Target protospacer sequences analyzed in this study.

Name	Sequence (5' → 3')	Analysis method
Can1-1	ATACTAAT$C_{-12}C_{-11}$ATGCCGCCAGTGG	canavanine selection
Can1-2	GCAAATTC_{-13}AAATATTTACGTTGG	canavanine selection
Can1-3	ACGT$C_{-16}C_{-15}$AAAATTGAATGACTTGG	canavanine selection, NGS
Can1-4	TTTC_{-17}AAGGTACTGAACTAGTTGG	canavanine selection
Can1-5	T$C_{-19}C_{-18}$AATAACGGAATCCAACTGGG	canavanine selection, NGS
Can1-6	GTTC_{-17}TC_{-15}AATAGCAATAATAAAGG	NGS
Can1-7	AAA$C_{-17}C_{-16}$AATACATGTAACCATTGG	NGS
Can1-8	TTGTT$C_{-15}C_{-14}C_{-13}$TGTCAAATATTACGG	NGS
Can1-9	C_{-20}ATATTGGTATGATTGCCCTTGG	canavanine selection
Can1-10	G$C_{-19}C_{-18}C_{-17}$TGGAACTTAGTGTAGTTGG	NGS
Can1-11	TTC_{-18}TC_{-16}TATGGAGGATGGCATAGG	NGS
Can1-12	TG$C_{-18}C_{-17}$TC_{-15}AATGTCTCTTCTATCGG	NGS
(C)$_{9}$ *motif*	CTTCCCCC$C_{-16}C_{-15}C_{-14}C_{-13}$ATGTTCCGAGATCGG	NGS
(C)$_{19}$ *motif*	$C_{-27}C_{-26}C_{-25}C_{-24}C_{-23}C_{-22}C_{-21}C_{-20}C_{-19}C_{-18}C_{-17}C_{-16}C_{-15}C_{-14}C_{-13}C_{-12}C_{-11}C_{-10}C_{-9}$GCATCAACTGG	NGS
(C)$_{8}$ *motif*	TATA$C_{-20}C_{-19}C_{-18}C_{-17}C_{-16}C_{-15}C_{-14}C_{-13}$TATATGGTAAAAAGG	NGS

Target Cs of editing are shown in red, with a subscript number indicating the position relative to the PAM. The PAM sequence is shown in blue.
